# Supplementary material for: Antibody affinity and cross-variant neutralization of SARS-CoV-2 Omicron BA.1, BA.2 and BA.3 following third mRNA vaccination
Source: Nat Commun. 2022 Aug 8;13:4617. doi: 10.1038/s41467-022-32298-w (PMC9358642; doi:10.1038/s41467-022-32298-w)
Supplement: Supplementary file 1 — Supplementary Information [file 41467_2022_32298_MOESM1_ESM.pdf]

Supplementary Table S1: SARS-CoV-2 variants mutations introduced in the spike plasmid for production of SARS-CoV-2 pseudovirions for analysis in PsVNA.

SARS-CoV-2 variant	Mutations constructed in the spike plasmids
**Omicron (BA.1/ B.1.1.529)**	A67V, H69-70del, T95I, G142D, V143-145del, Y145D, N211del, L212I, ins214EPE, G339D, S371L, S373P, S375F, K417N, N440K, G446S, S477N, T478K, E484A, Q493R, G496S, Q498R, N501Y, Y505H, T547K, D614G, H655Y, N679K, P681H, N764K, D796Y, N856K, Q954H, N969K, L981F
**Omicron (BA.2)**	T19I, delL24, delP25, delP26, A27S, G142D, V213G, G339D, S371F, S373P, S375F, T376A, D405N, R408S, K417N, N440K, S477N, T478K, E484A, Q493R, Q498R, N501Y, Y505H, D614G, H655Y, N679K, P681H, N764K, D796Y, Q954H, N969K
**Omicron (BA.3)**	A67V, del69-70, del142-144, Y145D, del211, L212I, G339D, S371F, S373P, S375F, D405N, K417N, N440K, G446S, S477N, T478K, E484A, Q493R, Q498R, N501Y, Y505H, D614G, H655Y, N679K, P681H, N764K, D796Y, Q954H, N969K

Supplementary Table S2a: Demographic and vaccination information of naïve adults.							
Naïve Adults	2nd dose vaccine	3rd dose vaccine					# days between COVID-19 and 1st vaccine dose
Sample Number	Type	Type	Sex	Race	Ethnicity	BMI	
N-1	Moderna	Pfizer	F	White	Not Hispanic or Latino	25.4	N/A
N-2	Moderna	Moderna	F	White	Not Hispanic or Latino	32.4	N/A
N-3	Pfizer	Pfizer	F	White	Not Hispanic or Latino	27.9	N/A
N-4	Pfizer	Pfizer	F	White	Not Hispanic or Latino	24.9	N/A
N-5	Pfizer	Moderna	F	White	Not Hispanic or Latino	22.2	N/A
N-6	Pfizer	Pfizer	F	White	Not Hispanic or Latino	44.4	N/A
N-7	Pfizer	Pfizer	F	White	Not Hispanic or Latino	31.9	N/A
N-8	Pfizer	Pfizer	F	White	Not Hispanic or Latino	30.3	N/A
N-9	Pfizer	Moderna	F	White	Not Hispanic or Latino	26.9	N/A
N-10	Pfizer	Pfizer	F	White	Not Hispanic or Latino	20.4	N/A
N-11	Pfizer	Pfizer	F	White	Not Hispanic or Latino	26	N/A
N-12	Pfizer	Moderna	F	White	Not Hispanic or Latino	24.1	N/A
N-13	Pfizer	Pfizer	F	White	Not Hispanic or Latino	29.1	N/A
N-14	Pfizer	Pfizer	M	White	Not Hispanic or Latino	35.6	N/A
N-15	Pfizer	Pfizer	M	White	Not Hispanic or Latino	27.2	N/A
N-16	Pfizer	Pfizer	M	White	Not Hispanic or Latino	28.5	N/A
N-17	Pfizer	Pfizer	M	White	Not Hispanic or Latino	30.8	N/A
N18	Moderna	Moderna	M	White	Not Hispanic or Latino	21	N/A
N-19	Pfizer	Pfizer	M	White	Not Hispanic or Latino	31.1	N/A
N-20	Moderna	Moderna	M	White	Not Hispanic or Latino	30.3	N/A
N-21	Moderna	Moderna	M	White	Not Hispanic or Latino	22.5	N/A
N-22	Pfizer	Pfizer	M	White	Not Hispanic or Latino	21.4	N/A
N-23	Moderna	Moderna	M	White	Not Hispanic or Latino	26.4	N/A
N-24	Pfizer	Pfizer	F	White	Not Hispanic or Latino	32.9	N/A
N-25	Pfizer	Pfizer	F	White	Not Hispanic or Latino	30.7	N/A
N-26	Pfizer	Pfizer	M	White	Not Hispanic or Latino	25.5	N/A
N-27	Moderna	Moderna	F	White	Not Hispanic or Latino	28	N/A
N-28	Pfizer	Pfizer	F	White	Not Hispanic or Latino	27.2	N/A
N-29	Moderna	Moderna	F	White	Not Hispanic or Latino	21	N/A
N-30	Pfizer	Pfizer	F	White	Not Hispanic or Latino	20.5	N/A

N-31	Pfizer	Pfizer	F	White	Not Hispanic or Latino	23.6	N/A
N-32	Pfizer	Pfizer	M	White	Not Hispanic or Latino	25.1	N/A
N-33	Pfizer	Pfizer	F	White	Not Hispanic or Latino	33.4	N/A
N-34	Moderna	Moderna	M	White	Not Hispanic or Latino	39.7	N/A
N-35	Pfizer	Moderna	F	White	Not Hispanic or Latino	29.9	N/A
N-36	Pfizer	Pfizer	F	White	Not Hispanic or Latino	25	N/A
N-37	Pfizer	Pfizer	F	White	Not Hispanic or Latino	27.7	N/A
N-38	Pfizer	Pfizer	F	White	Not Hispanic or Latino	28.1	N/A
N-39	Pfizer	Pfizer	F	White	Not Hispanic or Latino	22	N/A
N-40	Pfizer	Pfizer	M	White	Not Hispanic or Latino	19.2	N/A
N-41	Pfizer	Pfizer	F	White	Not Hispanic or Latino	24.3	N/A
N-42	Pfizer	Pfizer	F	White	Not Hispanic or Latino	24.8	N/A
N-43	Pfizer	Pfizer	F	White	Not Hispanic or Latino	29.3	N/A
N-44	Pfizer	Pfizer	F	White	Not Hispanic or Latino	26.3	N/A
N-45	Pfizer	Pfizer	F	White	Not Hispanic or Latino	25.9	N/A
N-46	Pfizer	Pfizer	F	White	Not Hispanic or Latino	21.9	N/A
N-47	Pfizer	Pfizer	F	White	Not Hispanic or Latino	27.7	N/A
N-48	Pfizer	Pfizer	M	White	Not Hispanic or Latino	29	N/A
N-49	Pfizer	Moderna	M	White	Not Hispanic or Latino	23.5	N/A
N-50	Pfizer	Moderna	M	White	Not Hispanic or Latino	23.7	N/A
N-51	Pfizer	Pfizer	M	White	Not Hispanic or Latino	21.4	N/A

Supplementary Table S2b: Demographic and vaccination information of COVID-19 adults.							
Covid Adults	2nd dose vaccine	3rd dose vaccine					# days between COVID-19 and 1st vaccine dose
Sample Number	Type	Type	Sex	Race	Ethnicity	BMI	
C-1	Pfizer	Pfizer	F	White	Not Hispanic or Latino	26.1	181
C-2	Pfizer	Pfizer	F	White	Hispanic or Latino	30.4	25
C-3	Pfizer	Pfizer	F	White	Not Hispanic or Latino	28.8	65
C-4	Pfizer	Pfizer	F	White	Not Hispanic or Latino	37.5	190
C-5	Pfizer	Pfizer	F	White	Not Hispanic or Latino	27.5	159
C-6	Pfizer	Pfizer	F	White	Not Hispanic or Latino	43.5	52
C-7	Moderna	Moderna	F	White	Not Hispanic or Latino	29.1	34
C-8	Pfizer	Pfizer	F	White	Not Hispanic or Latino	22.9	45
C-9	Pfizer	Pfizer	F	White	Not Hispanic or Latino	30.7	80
C-10	Pfizer	Pfizer	F	White	Not Hispanic or Latino	28.1	106
C-11	Pfizer	Pfizer	F	White	Not Hispanic or Latino	27.7	195
C-12	Pfizer	Pfizer	F	White	Not Hispanic or Latino	30.3	210
C-13	Pfizer	Pfizer	F	White	Not Hispanic or Latino	36.3	N/A
C-14	Pfizer	Pfizer	F	Black	Not Hispanic or Latino	31.5	54
C-15	Pfizer	Pfizer	F	White	Not Hispanic or Latino	41.2	55
C-16	Pfizer	Pfizer	F	White	Not Hispanic or Latino	30.1	17
C-17	Pfizer	Moderna	M	White	Not Hispanic or Latino	26.5	36
C-18	Pfizer	Pfizer	M	White	Not Hispanic or Latino	40.5	97
C-19	Pfizer	Pfizer	M	White	Not Hispanic or Latino	34.2	N/A
C-20	Pfizer	Pfizer	M	White	Not Hispanic or Latino	32.3	263
C-21	Moderna	Moderna	F	White	Not Hispanic or Latino	N/A	35
C-22	Pfizer	Pfizer	M	White	Not Hispanic or Latino	30.8	86
C-23	Moderna	Pfizer	M	White	Not Hispanic or Latino	29.2	N/A
C-24	Pfizer	Pfizer	M	White	Hispanic or Latino	31.3	153
C-25	Pfizer	Pfizer	M	White	Not Hispanic or Latino	23.3	57
C-26	Pfizer	Pfizer	F	White	Not Hispanic or Latino	27.2	186
C-27	Pfizer	Pfizer	F	White	Not Hispanic or Latino	35	196
C-28	Pfizer	Pfizer	M	White	Not Hispanic or Latino	25.7	81
C-29	Pfizer	Pfizer	F	White	Hispanic or Latino	49.5	108
C-30	Moderna	Moderna	M	White	Not Hispanic or Latino	32.2	39
C-31	Pfizer	Pfizer	F	White	Not Hispanic or Latino	21.7	N/A

Supplementary Figure S1

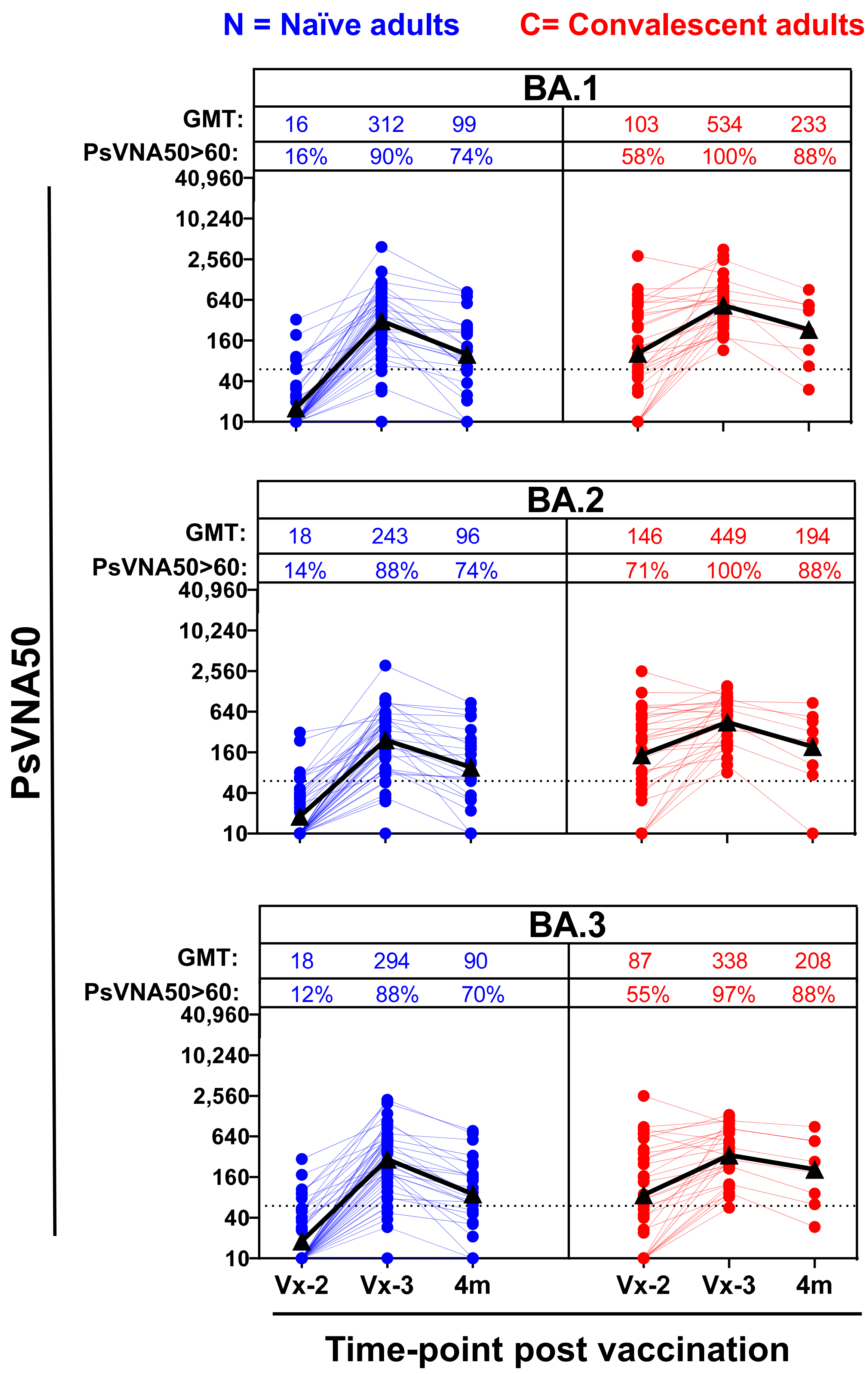

Supplementary Figure S1: Neutralizing antibody titers of longitudinal post-vaccination serum from in naïve and convalescent adults against SARS-CoV-2 WA1 and Omicron subvariants. SARS-CoV-2 neutralizing antibody titers in post-vaccination serum of 50 unexposed naïve adults (N; in blue) and 31 COVID-19 survivors (C; in red) as determined by pseudovirus neutralization assay (PsVNA) in 293-ACE2-TMPRSS2 cells with SARS-CoV-2 Omicron subvariants BA.1, BA.2 and BA.3. PsVNA50 (50% neutralization titer) titers of post-2nd (Vx-2; at 1 month post-second dose) or at two different time-points following third vaccination, either at 1 month post-third dose (Vx-3) or 4-months post-third dose (4m; for 27 unexposed naïve adults and 8 convalescent adults) serum samples against the vaccine-matched WA1 strain. Geometric mean PsVNA50 titers (GMT) are shown as black triangles and are presented for each vaccination time-point on top of the panel. Each data point represents an individual sample (circles) and indicates the 50% neutralization titer obtained with each sample. The horizontal dashed line indicates the seropositive cut-off for the neutralization titers (PsVNA50 of 60). Percent seropositivity (%S) for each group was calculated as number of seropositive samples in the group divided by total number of samples x 100 in the group. All PsVNA experiments were performed in duplicate and the researchers performing the assay were blinded to sample identity. The variations for duplicate runs were <6%. The data shown are average values of two experimental runs. Differences between different time-points were analyzed by lme4 and emmeans packages in R using Tukey's pairwise multiple comparison test that controlled for age, sex and BMI as covariates and the two-sided statistically significant p-values are shown. Non-significant p-values ($p > 0.05$) are not shown.

Supplementary Figure S2

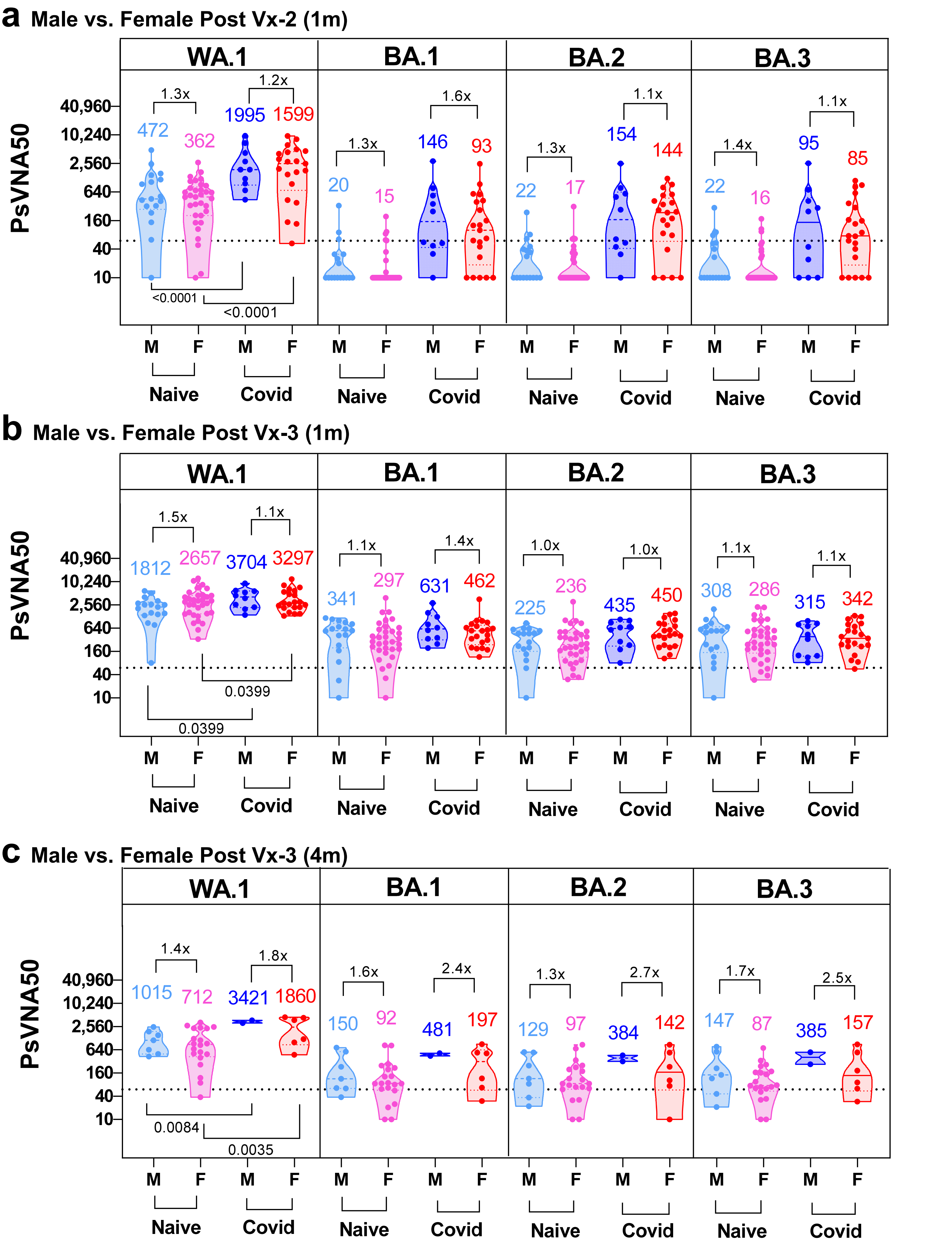

Supplementary Figure S2: SARS-CoV-2 neutralization against SARS-CoV-2 WA1 and Omicron subvariants following SARS-CoV-2 mRNA vaccination in males vs females. Box and whisker plots show comparison of SARS-CoV-2 neutralizing antibody titers in post-vaccination serum of unexposed naïve males (M in cyan; N=17) vs females (F in pink; N=33) and COVID-19 males (M in navy blue; N=10) vs females (F in red; N=21) as determined by pseudovirus neutralization assay (PsVNA) in 293-ACE2-TMPRSS2 cells with SARS-CoV-2 Omicron subvariants BA.1, BA.2 and BA.3. PsVNA50 (50% neutralization titer) titers of post-2nd (Vx-2; at 1 month post-second dose in panel a) or at two different time-points following third vaccination, either at 1 month post-third dose (Vx-3; panel b) or 4-months post-third dose (4m; in panel c) serum samples against the vaccine-matched WA1 strain. Geometric mean PsVNA50 titers (GMT) are presented for each group on top of the panel. Each data point represents an individual sample (circles) and indicates the 50% neutralization titer obtained with each sample. The horizontal dashed line indicates the seropositive cut-off for the neutralization titers (PsVNA50 of 60). All PsVNA experiments were performed in duplicate and the researchers performing the assay were blinded to sample identity. The variations for duplicate runs were <6%. The data shown are average values of two experimental runs. Differences between different time-points were analyzed by lme4 and emmeans packages in R using Tukey's pairwise multiple comparison test that controlled for age, sex and BMI as covariates and the two-sided statistically significant p-values are shown. Non-significant p-values ($p > 0.05$) are not shown.

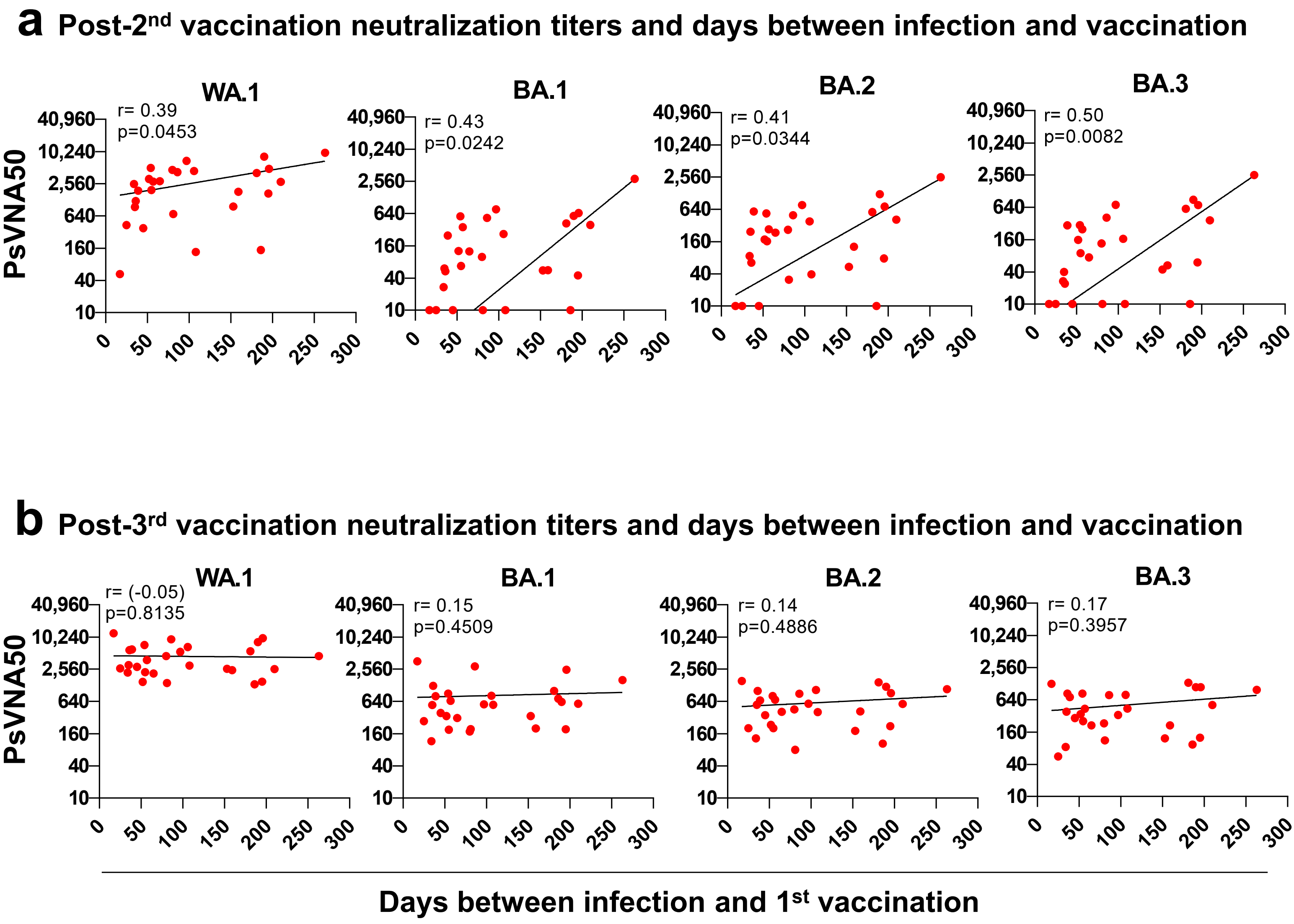

Supplementary Figure S3: Relationship of post-vaccination SARS-CoV-2 serum neutralizing antibodies in COVID convalescent adults with time-interval between infection and first vaccination. Correlation analysis between serum PsVNA50 neutralization antibody titers generated following second (a) and third vaccination (b) of COVID exposed (n=31) adults against vaccine-matched SARS-CoV-2 WA1 and Omicron BA.1, BA.2, BA.3 and time-interval between SARS-CoV-2 infection and first mRNA vaccination in these adults. Correlation analysis was performed using non-linear regression model and associated Spearman's correlation coefficients (r) and regression significance (p) are shown for two-sided statistical test.

Supplementary Figure S4

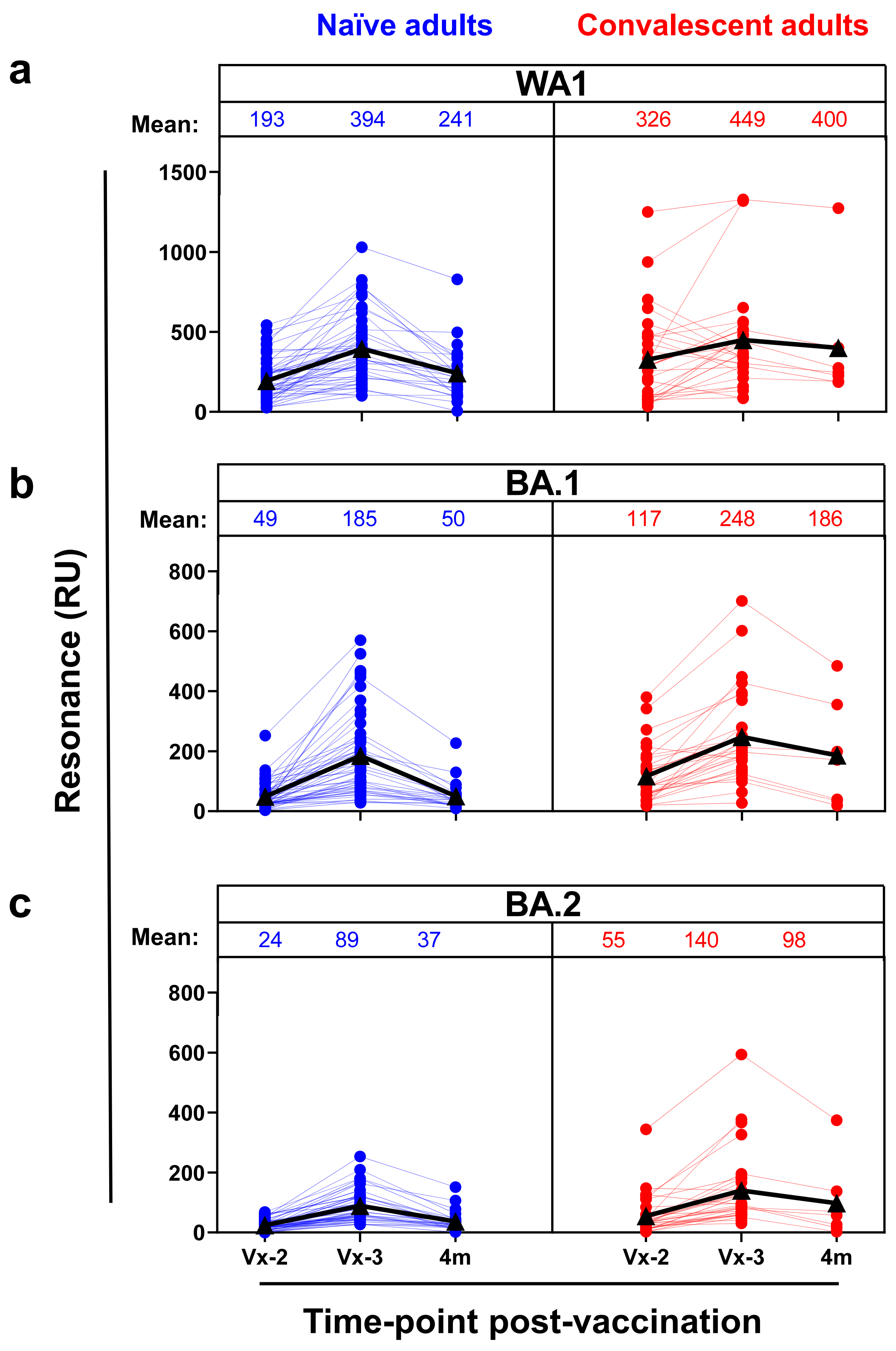

Supplementary Figure S4. Longitudinal binding antibodies against spike of SARS-CoV-2 WA1 or Omicron BA.1 and BA.2 variants following second and third vaccination in naïve and convalescent adults.

(a-c) Binding antibody to spike protein of vaccine-homologous SARS-CoV-2 WA1/2020 (a) or Omicron BA.1 variant (b) and BA.2 variant (c) in serum from 50 unexposed naïve adults (in blue) and 31 COVID-19 survivors (in red) following 2nd (Vx-2; at 1 month post-second dose) or at two different time-points following third vaccination, either at 1 month post-third dose (Vx-3) or 4-months post-third dose (D116; for 27 unexposed naïve adults and 8 convalescent adults) serum. The mean values are color coded by each cohort are shown. All SPR experiments were performed twice and the researchers performing the assay were blinded to sample identity. The variation for each sample in duplicate SPR runs was <4%. The data shown are the average value of two experimental runs. Statistical differences between different time-points were analyzed by lme4 and emmeans packages in R using Tukey's pairwise multiple comparison test that controlled for age, sex and BMI as covariate and the two-sided statistically significant p-values are shown. Non-significant p-values (*p >0.05*) are not shown.

Supplementary Figure S5

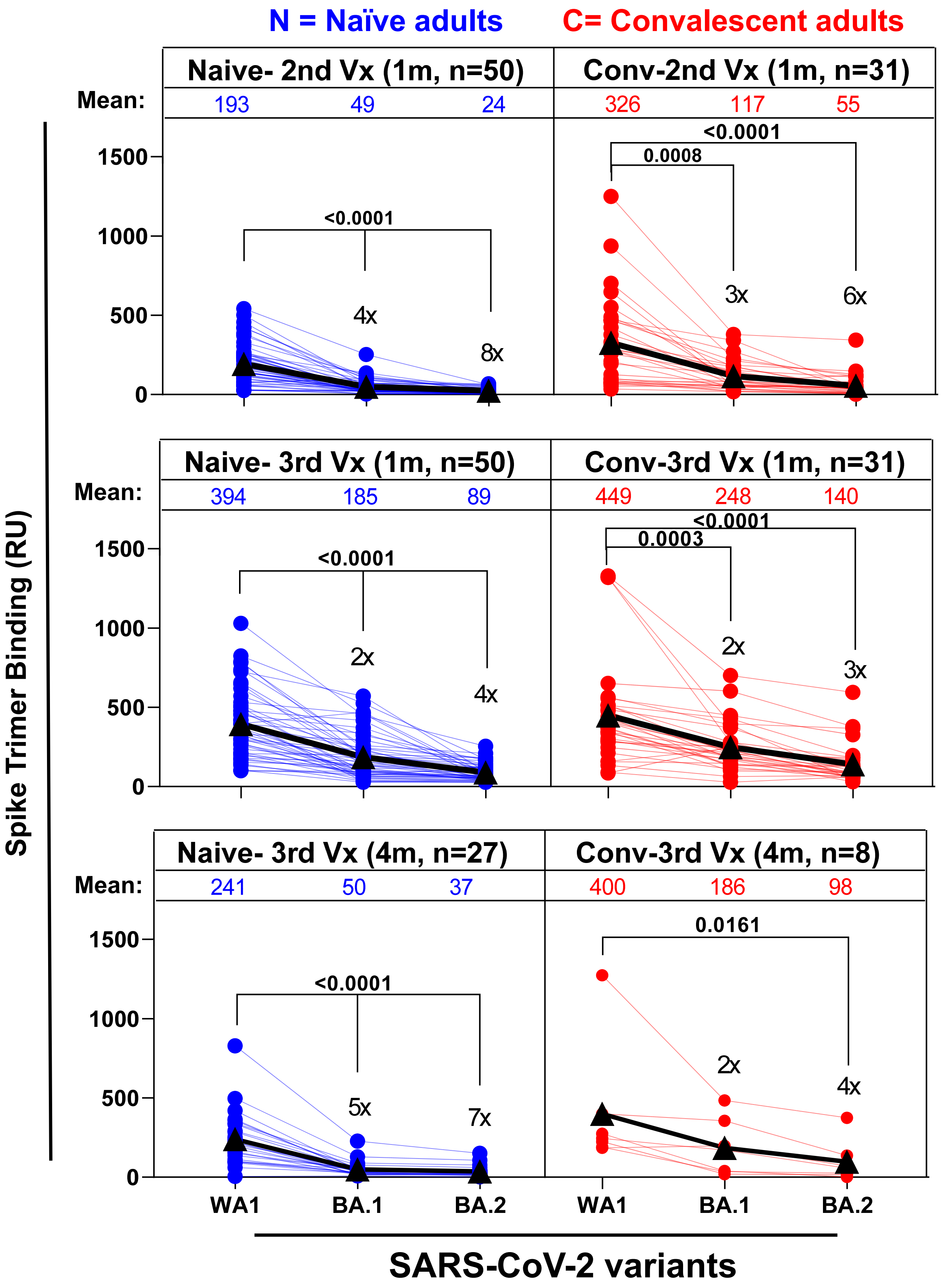

Supplementary Figure S5: Binding antibodies following second or third vaccination to SARS-CoV-2 spike of WA1 vs Omicron BA.1 vs BA.2 by SPR. Total antibody binding (determined by maximum resonance units, Max RU) of 1:10 diluted serum on 1 month after second (Vx-2) or third mRNA vaccination (Vx-3) or 4 months post-3rd vaccination (4m) samples of unexposed naïve (in blue) adults and COVID-19 survivors (in red) to purified trimeric spike of WA1 vs Omicron subvariants BA.1 and BA.2 by SPR. Mean antibody binding values are shown as black triangles and are presented for each spike. The fold-reduction in binding antibodies to BA.1 or BA.2 spike compared with WA1 spike are shown. All SPR experiments were performed in duplicate, and the researchers performing the assay were blinded to sample identity. The variations for duplicate runs of SPR were <5%. The data shown are average values of two experimental runs. Statistical differences of SARS-CoV-2 BA.1 or BA.2 spike compared with WA1 spike were analyzed by lme4 and emmeans packages in R using Tukey's pairwise multiple comparison test that controlled for age, sex and BMI as covariate and the two-sided statistically significant p-values are shown. Non-significant p-values (*p >0.05*) are not shown.

Supplementary Figure S6

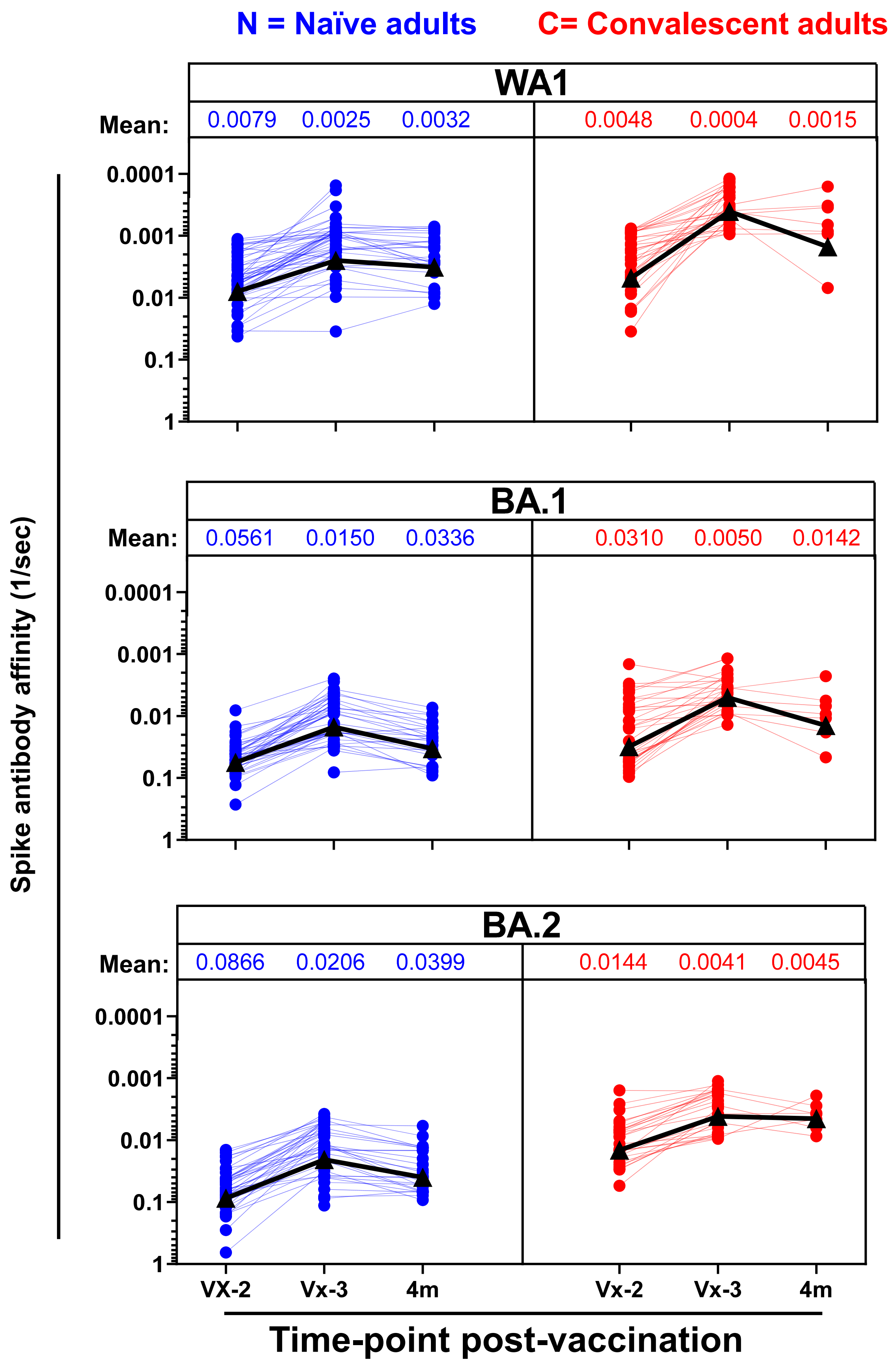

Supplementary Figure S6: Antibody affinity maturation following second or third vaccination to SARS-CoV-2 spike of WA1 vs Omicron BA.1 vs BA.2 in naïve vs convalescent individuals. Antibody affinity maturation (as measured by dissociation off-rate per sec) of human serum were measured on 1 month after second (Vx-2) or third mRNA vaccination (Vx-3) and 4 months post-3rd vaccination (4m) samples of unexposed naïve (in blue) adults and COVID-19 survivors (in red) to purified trimeric spike of WA1 or Omicron subvariants BA.1 or BA.2 by SPR. Antibody off-rate constants that describe the fraction of antibody-antigen complexes decaying per second were determined directly from the serially diluted post-vaccination sample interaction with SARS-CoV-2 spike proteins using SPR in the dissociation phase as described in Materials and Methods. Off-rate was calculated and shown only for the sample time points that demonstrated a measurable (>10RU) antibody binding in SPR. Antibody affinity was not determined for those serum whose spike-binding antibodies were <10RU. Mean antibody off-rate values are shown as black triangles and are presented for each spike. All SPR experiments were performed in duplicate, and the researchers performing the assay were blinded to sample identity. The variations for duplicate runs of SPR were <5%. The data shown are average values of two experimental runs. Statistical differences between time-points were analyzed by lme4 and emmeans packages in R using Tukey's pairwise multiple comparison test that controlled for age, sex and BMI as covariate and the two-sided statistically significant p-values are shown. Non-significant p-values ($p > 0.05$) are not shown.

Supplementary Figure S7

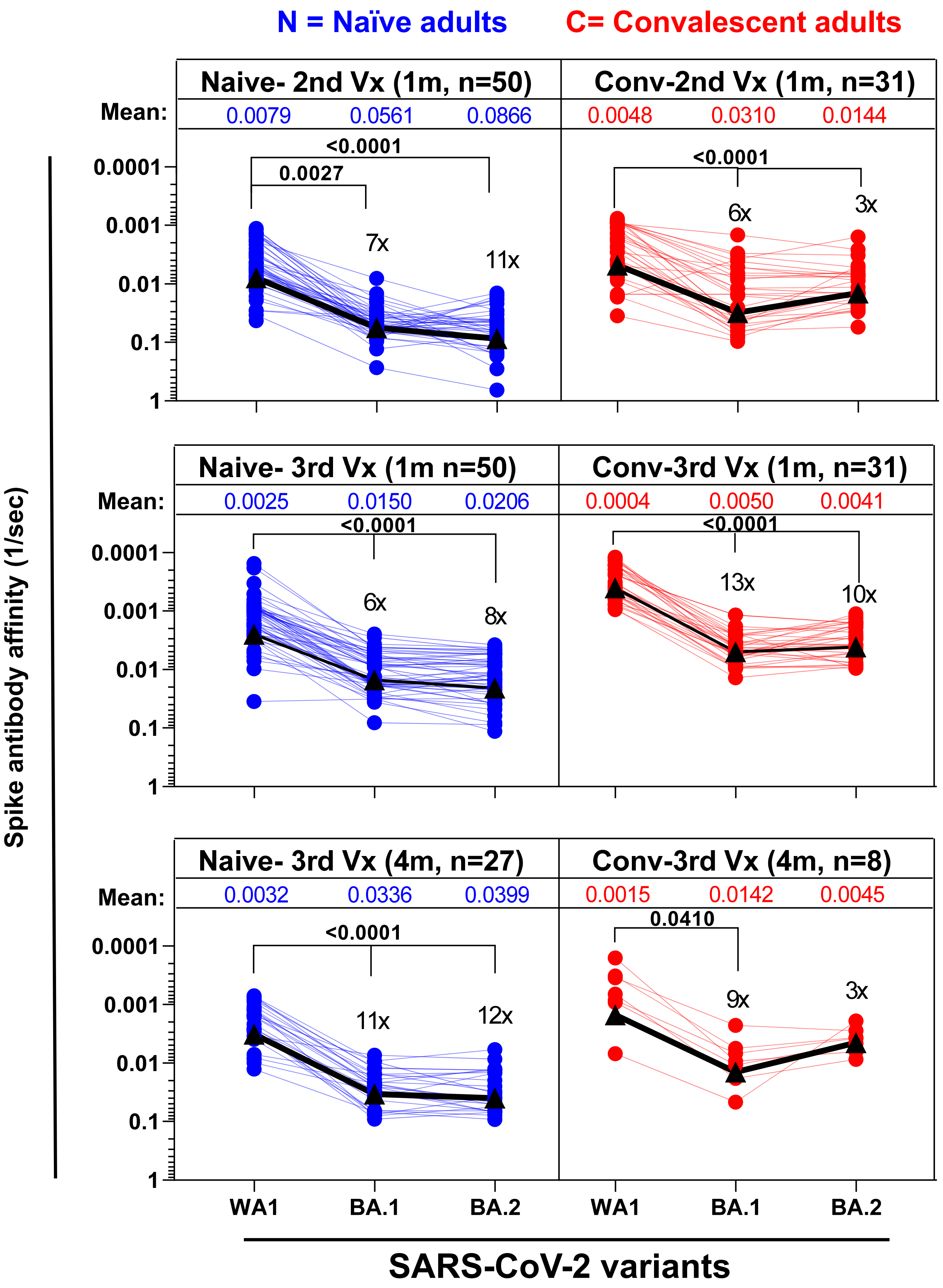

Supplementary Figure S7: Antibody affinity against spike protein of SARS-CoV-2 WA1 vs Omicron BA.1 vs BA.2 following second or third vaccination in naïve vs convalescent individuals. Antibody affinity (as measured by dissociation off-rate per sec) of human serum at 1 month after second (Vx-2) or third mRNA vaccination (Vx-3) or 4 months post-3rd vaccination (4m) samples of unexposed naïve (in blue) adults and COVID-19 survivors (in red) to purified trimeric spike of WA1 vs Omicron subvariants BA.1 vs BA.2 by SPR. Antibody off-rate constants that describe the fraction of antibody-antigen complexes decaying per second were determined directly from the serially diluted post-vaccination sample interaction with SARS-CoV-2 spike proteins using SPR in the dissociation phase as described in Materials and Methods. Off-rate was calculated and shown only for the sample time points that demonstrated a measurable (>10RU) antibody binding in SPR. Antibody affinity was not determined for those serum whose spike-binding antibodies were <10RU. Mean antibody off-rate values are shown as black triangles and are presented for each spike. The fold-difference in antibody off-rate to BA.1 or BA.2 spike compared with WA1 spike is shown along with p-values. All SPR experiments were performed in duplicate, and the researchers performing the assay were blinded to sample identity. The variations for duplicate runs of SPR were <5%. The data shown are average values of two experimental runs. Statistical differences of SARS-CoV-2 BA.1 or BA.2 spike compared with WA1 spike were analyzed by lme4 and emmeans packages in R using Tukey's pairwise multiple comparison test that controlled for age, sex and BMI as covariate and the two-sided statistically significant p-values are shown. Non-significant p-values ($p > 0.05$) are not shown.

Supplementary Figure S8

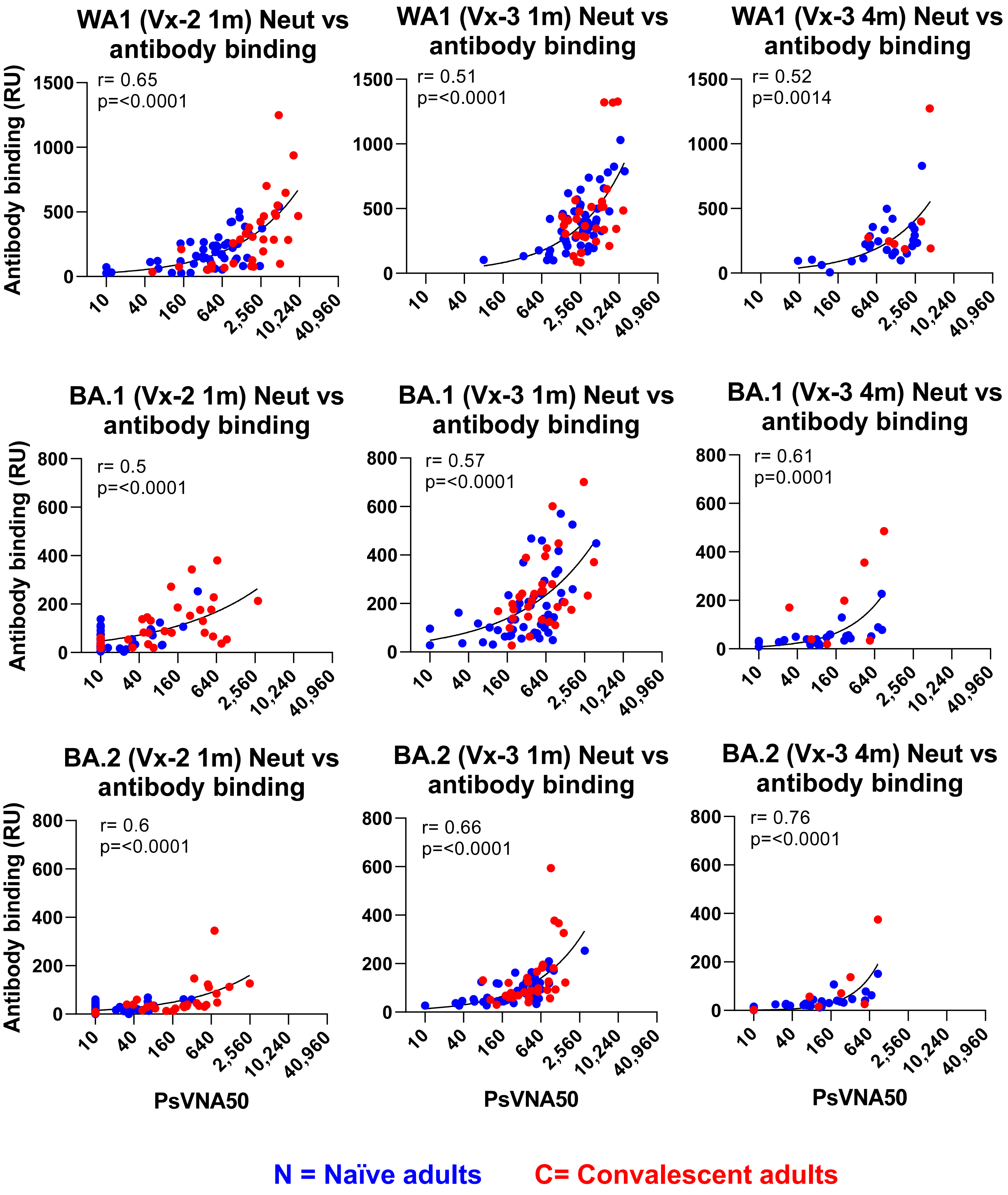

Supplementary Figure S8: Relationship of post-vaccination SARS-CoV-2 serum neutralizing antibodies in COVID convalescent and naïve adults with antibody binding against SARS-CoV-2 spike protein. Correlation analysis between serum PsVNA50 neutralization antibody titers generated following second and third vaccination of COVID exposed (n=31) and unexposed naïve (n=50) adults against vaccine-matched SARS-CoV-2 WA1 and Omicron BA.1 and BA.2, and antibody binding to spike protein of corresponding SARS-CoV-2 strain. Correlation analysis was performed using non-linear regression model and associated Spearman’s correlation coefficients (r) and regression significance (p) are shown for two-sided statistical test.
