# Supplementary material for: Antibody affinity and cross-variant neutralization of SARS-CoV-2 Omicron BA.1, BA.2 and BA.3 following third mRNA vaccination
Source: Nat Commun. 2022 Aug 8;13:4617. doi: 10.1038/s41467-022-32298-w (PMC9358642; doi:10.1038/s41467-022-32298-w)
Supplement: Supplementary file 3 — Reporting Summary [file 41467_2022_32298_MOESM3_ESM.pdf]

Corresponding author(s): Surender Khurana
Last updated by author(s): Jul 19, 2022

Reporting Summary

Nature Research wishes to improve the reproducibility of the work that we publish. This form provides structure for consistency and transparency in reporting. For further information on Nature Research policies, see our Editorial Policies and the Editorial Policy Checklist.

Statistics

For all statistical analyses, confirm that the following items are present in the figure legend, table legend, main text, or Methods section.

n/a	Confirmed	
☐	☒	The exact sample size (*n*) for each experimental group/condition, given as a discrete number and unit of measurement
☐	☒	A statement on whether measurements were taken from distinct samples or whether the same sample was measured repeatedly
☐	☒	The statistical test(s) used AND whether they are one- or two-sided<br>*Only common tests should be described solely by name; describe more complex techniques in the Methods section.*
☐	☒	A description of all covariates tested
☐	☒	A description of any assumptions or corrections, such as tests of normality and adjustment for multiple comparisons
☐	☒	A full description of the statistical parameters including central tendency (e.g. means) or other basic estimates (e.g. regression coefficient) AND variation (e.g. standard deviation) or associated estimates of uncertainty (e.g. confidence intervals)
☐	☒	For null hypothesis testing, the test statistic (e.g. *F*, *t*, *r*) with confidence intervals, effect sizes, degrees of freedom and *P* value noted<br>*Give P values as exact values whenever suitable.*
☒	☐	For Bayesian analysis, information on the choice of priors and Markov chain Monte Carlo settings
☒	☐	For hierarchical and complex designs, identification of the appropriate level for tests and full reporting of outcomes
☐	☒	Estimates of effect sizes (e.g. Cohen's *d*, Pearson's *r*), indicating how they were calculated

Our web collection on statistics for biologists contains articles on many of the points above.

Software and code

Policy information about availability of computer code

Data collection	Antibody data was collected in MS Excel version 16.57. BioRad ProteON Manager software (Version 3.1.0) was used to collect antibody binding data from SPR machine (www.Biorad.com)
Data analysis	BioRad ProteON Manager software (Version 3.1.0) for antibody binding analysis from SPR machine (www.Biorad.com). Neutralizing Antibody titers were calculated using Prism 9.3.1 (GraphPad Software). Statistical analysis were performed using Ime4 and emmeans packages in R statistical software (RStudio version 1.1.463).

For manuscripts utilizing custom algorithms or software that are central to the research but not yet described in published literature, software must be made available to editors and reviewers. We strongly encourage code deposition in a community repository (e.g. GitHub). See the Nature Research guidelines for submitting code & software for further information.

Data

Policy information about availability of data

All manuscripts must include a data availability statement. This statement should provide the following information, where applicable:

- Accession codes, unique identifiers, or web links for publicly available datasets
- A list of figures that have associated raw data
- A description of any restrictions on data availability

All data are shown in the manuscript figures and supplementary information. The complete dataset for this study are provided in the Source Data file.

April 2020

Field-specific reporting

Please select the one below that is the best fit for your research. If you are not sure, read the appropriate sections before making your selection.

☒ Life sciences ☐ Behavioural & social sciences ☐ Ecological, evolutionary & environmental sciences

For a reference copy of the document with all sections, see nature.com/documents/nr-reporting-summary-flat.pdf

Life sciences study design

All studies must disclose on these points even when the disclosure is negative.

Sample size	All available samples were analyzed in this study
Data exclusions	No data was excluded
Replication	Neutralization and SPR were performed twice by independent researchers in the lab. The replications were successful. The variation in duplicate experimental runs was <9% for neutralization and <6% for SPR.
Randomization	All samples from the vaccinated adults were analyzed in this study. The study was non-randomized performed during the pandemic. Initially, no patient information was provided, and all the immune analyses were conducted blindly by the researcher's performing the assays. The participants were assigned in each experimental group based on their prior SARS-CoV-2 history, as either naive or convalescent.
Blinding	Experiments were performed by different investigators, who were blinded to sample identity.

Reporting for specific materials, systems and methods

We require information from authors about some types of materials, experimental systems and methods used in many studies. Here, indicate whether each material, system or method listed is relevant to your study. If you are not sure if a list item applies to your research, read the appropriate section before selecting a response.

Materials & experimental systems

n/a	Involved in the study	
☒	☐	Antibodies
☐	☒	Eukaryotic cell lines
☒	☐	Palaeontology and archaeology
☒	☐	Animals and other organisms
☐	☒	Human research participants
☒	☐	Clinical data
☒	☐	Dual use research of concern

Methods

n/a	Involved in the study	
☒	☐	ChIP-seq
☒	☐	Flow cytometry
☒	☐	MRI-based neuroimaging

Eukaryotic cell lines

Policy information about cell lines

Cell line source(s)	Lenti-X- 293T cells were obtained from Takara Bio (Cat. No. 632180). 293_ACE2_TMPRSS2 cell line was generated and sourced from the lab of Carol Weiss at FDA [see reference Neerukonda, S.N. et al. (2021) PLoS One 16, e0248348].
Authentication	Cell lines were checked for expression of ACE2 and validated by FACS analysis. None of the cell lines were authenticated by karyotyping or other genomic techniques. Reference: Neerukonda, S.N. et al. Establishment of a well-characterized SARS-CoV-2 lentiviral pseudovirus neutralization assay using 293T cells with stable expression of ACE2 and TMPRSS2. PLoS One 16, e0248348 (2021).
Mycoplasma contamination	Negative for Mycoplasma
Commonly misidentified lines (See ICLAC register)	No misidentified cell lines were used in the study.

Human research participants

Policy information about studies involving human research participants

Population characteristics	Participants in this study were all adults. Individuals of any gender that recieved COVID-19 vaccination were eligible for the study. Sex and/or gender was considered in the study design and was determined based on self-report by the study participants. All participants reported to be binary (either Male or Female). Covariates included convalescent individuals who were exposed to SARS-CoV-2 but not hospitalized (had confirmed SARS-CoV-2 infection between March – November 2020) as well as naïve healthy adults. None of the participants reported SARS-CoV-2 breakthrough infection following vaccination.
Recruitment	All SARS-CoV-2 vaccinees were eligible without any specific selection criteria and no selection bias or any other apparent bias. Samples were collected from adults following informed consent to participate in the study during the pandemic. Adult participants were recruited and enrolled in the SPARTA (SARS2 Seroprevalence and Respiratory Tract Assessment) program in Athens, GA (USA).
Ethics oversight	Samples were obtained from participants enrolled in the SPARTA (SARS2 Seroprevalence and Respiratory Tract Assessment) program in Athens, GA (USA) with written informed consent. The study procedures, informed consent, and data collection documents were reviewed and approved by the WIRB-Copernicus Group Institutional Review Board (WCG IRB #202029060) and the University of Georgia. All patients provided written informed consent. Consent on this study included agreement for the use of remnant material for additional immunological assays at the time of study enrollment. Samples were tested in different antibody assays with approval from the U.S. Food and Drug Administration's Research Involving Human Subjects Committee (FDA-RIHSC) under exemption protocol '252-Determination- CBER-2020-08-19.

Note that full information on the approval of the study protocol must also be provided in the manuscript.
